# Association between equivalized annual household income and regular medical visits for hypertensive patients since the COVID-19 outbreak

**DOI:** 10.1038/s41440-024-02067-x

**Published:** 2025-01-08

**Authors:** Maya Toyama, Michihiro Satoh, Hideaki Hashimoto, Yutaro Iwabe, Takahito Yagihashi, Shingo Nakayama, Takahisa Murakami, Naoki Nakaya, Hirohito Metoki, Atsushi Hozawa, Takahiro Tabuchi

**Affiliations:** 1 Department of Preventive Medicine and Epidemiology, Tohoku Medical Megabank Organization, Tohoku University, Sendai, Japan; 2 Division of Public Health, Hygiene and Epidemiology, Faculty of Medicine, Tohoku Medical and Pharmaceutical University, Sendai, Japan; 3 Department of Nephrology, Self-Defense Forces Sendai Hospital, Sendai, Japan; 4 Department of Pharmacy, Tohoku Medical and Pharmaceutical University Hospital, Sendai, Japan; 5 Division of Nephrology and Endocrinology, Faculty of Medicine, Tohoku Medical and Pharmaceutical University, Sendai, Japan; 6 Center for Clinical Research Promotion and Development, Tohoku Medical and Pharmaceutical University Hospital, Sendai, Japan; 7 Department of Cerebrovascular Medicine, Konan Hospital, Sendai, Japan; 8 Division of Aging and Geriatric Dentistry, Department of Rehabilitation Dentistry, Tohoku University Graduate School of Dentistry, Sendai, Japan; 9 Tohoku Institute for Management of Blood Pressure, Sendai, Japan; 10 Division of Epidemiology, School of Public Health, Tohoku University Graduate School of Medicine, Sendai, Japan

**Keywords:** Health inequities, Socioeconomic factors, COVID-19, Hypertension, Epidemiology

## Abstract

Previous studies have shown an increase in blood pressure during the coronavirus disease 2019 (COVID-19) pandemic even among patients receiving antihypertensive treatment. This study aims to evaluate the association between equivalized annual household income and refraining from regular medical visits for hypertensive patients since the COVID-19 outbreak. We analyzed data from the Japan COVID-19 and Society Internet Survey (JACSIS), including 2832 hypertensive patients aged 20–79 years from the 2020 survey and at least one survey between 2021 and 2023. They were categorized into lower-income (<median of ¥3,182,000) and higher-income (≥median) groups. Refraining from regular medical visits was defined as not attending scheduled medical visits for hypertension in the past two months. Poisson regression or generalized linear mixed models were used, inverse probability weighted for Internet survey selection. After weighting, the mean age was 64.8 ± 10.3 years and 63.7% were men. In 2020, the proportion of hypertensive patients refraining from regular medical visits after weighting was 19.6% in the lower-income group and 8.8% in the higher-income group, with an adjusted proportion ratio (95% confidence interval) of 1.86 (1.13–3.06) for the lower-income group compared with the higher-income group. After 2020, the proportion of those refraining from regular medical visits declined in all income groups, and the income-related differences disappeared. During the social restrictions due to the COVID-19 pandemic, hypertensive patients with lower equivalized annual household incomes were more likely to refrain from regular medical visits. Strategies to reduce income-related inequities in medical care utilization may be necessary for future public health crises.

**Figure:**
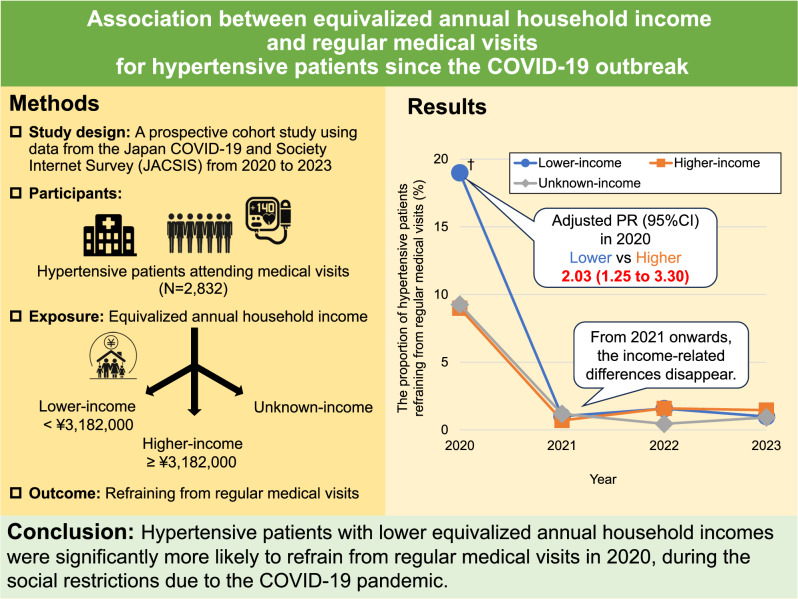
A nationwide Internet survey in Japan revealed hypertensive patients with lower equivalized annual household incomes were significantly more likely to refrain from regular medical visits in 2020, during the social restrictions due to the COVID-19 pandemic. Addressing income-related inequities in medical care utilization is crucial for future public health crises.

A nationwide Internet survey in Japan revealed hypertensive patients with lower equivalized annual household incomes were significantly more likely to refrain from regular medical visits in 2020, during the social restrictions due to the COVID-19 pandemic. Addressing income-related inequities in medical care utilization is crucial for future public health crises.

## Introduction

The Coronavirus disease 2019 (COVID-19) pandemic and associated public health measures disrupted the lives of people worldwide. Many countries imposed lockdowns and social restrictions to stop the spread of the infection at that time. In April 2020, the Japanese government also declared a state of emergency due to the nationwide spread of COVID-19 infection and the increasing number of deaths, urging citizens to stay home and avoid unnecessary outings. During the COVID-19 pandemic, there were several reports of delays in or refraining from medical care [1–3], and marked reductions in outpatient visits and hospital admissions [4, 5].

Socioeconomic status (SES) based on income, education, and employment has been shown to affect health problems [6]. Reducing these health inequities is a global public health priority [7]. People with lower SES have also reported poorer access to healthcare [8, 9]. Online surveys conducted in the US and Japan showed that during the COVID-19 pandemic, people with lower household incomes were more likely to delay or refrain from medical care [10, 11]. However, few studies have examined the association between income and medical care visits during the COVID-19 pandemic for specific diseases, and the association between the two after social restrictions due to the COVID-19 pandemic remains unknown.

Hypertension is a major risk factor for cardiovascular and chronic kidney diseases worldwide [12, 13]. Previous studies have shown a 1–2/0.5–1 mmHg increase in systolic/diastolic blood pressure after adjustment for covariates during the COVID-19 pandemic even among patients receiving antihypertensive treatment [14, 15]. The outpatient visits among hypertensive patients may have also decreased during the same period [4, 16], suggesting that the restricted access to medical care for hypertensive patients may have contributed to poorer blood pressure control [17]. Meanwhile, among hypertensive patients, no studies have investigated the social backgrounds of reductions in outpatient visits and how outpatient visit actions changed after social restrictions due to the pandemic.

This study focused on the medical care utilization of hypertensive patients and aimed to examine the association between household income and regular medical visits since the COVID-19 outbreak.

## Methods

### Study design

The current study analyzed data from the Japan COVID-19 and Society Internet Survey (JACSIS), a prospective, Internet-based, self-reported questionnaire survey conducted by a major Internet research agency (Rakuten Insight, INC) from 2020 to 2023. The JACSIS was designed to assess the impact of the COVID-19 pandemic on the health and social conditions in Japan. Questionnaires used for the 2020 baseline survey were distributed from August 25 to September 30, 2020, until the target sample numbers by sex, age, and prefecture category were achieved. Data were collected from a total of 28,000 respondents aged 15–79 years out of 2.2 million participants registered with Rakuten Insight, Inc. The research agency sent survey invitations to 224,389 candidates from these 2.2 million participants. A computer algorithm was used to facilitate the random sampling of participants for this survey. We set a target of 28,000 respondents to ensure enough participants in each age and sex group for accurate estimates while staying within our survey budget. The sample reflected the official demographic composition of Japan as of October 1, 2019, by age, sex, and prefecture categories. The 2021–2023 surveys were similarly conducted between September and October, respectively. We first cross-sectionally tested the association between income and the proportion of participants refraining from regular medical visits in 2020. Then, the change in the proportion of those refraining from regular medical visits after 2020 was assessed prospectively.

All procedures were conducted in accordance with the ethical standards of the Declaration of Helsinki. The study was reviewed and approved by the Research Ethics Committee of the Tohoku University Graduate School of Medicine (approved on June 27, 2024; Approval No. 2024-1-231) and Tohoku Medical and Pharmaceutical University (approved on June 6, 2023; Approval No. 2023-010).

### Participants

Of the 28,000 respondents, we excluded 2518 participants who provided invalid responses. These exclusions were performed to validate data quality and have been described in previous studies [10, 18–20]. From these, we selected 3414 participants who were currently attending medical visits for hypertension treatment. Additionally, 10 participants aged < 20 years at baseline were excluded because they were more likely to be financially dependent on their parents and the total number was small. We also excluded 572 participants who never participated in any of the surveys from 2021 to 2023. Finally, 2832 participants were included in the present analysis.

### Measurement of variables

#### Exposure

Information on participants’ annual household income was collected through the JACSIS questionnaires. We used equivalized annual household income in 2020 as the exposure variable. Equivalized annual household income was calculated by dividing annual household income by the square root of the number of household members to adjust for differences in household size [9, 10, 21–23]. We divided the participants into three groups based on the median equivalized annual household income: a lower-income group, below 3,182,000 yen (~31,820 US dollars in 2020); a higher-income group, at or above 3,182,000 yen; and an unknown-income group [10].

The definition of hypertensive patients attending medical visits was based on the question “Do you have a history of hypertension? (never/currently not, but had in the past/yes, currently (attending medical visits)/yes, currently (not attending medical visits))” with the response “yes, currently (attending medical visits).”

### Outcomes

Refraining from regular medical visits for hypertensive patients in each survey from 2020 to 2023 was used as the outcome.

The outcome in 2020 was obtained from the question, “Did you refrain from a regular medical visit between April and May 2020?” The corresponding question between 2021 and 2023 was, “Have you refrained from regular medical visits for hypertension in the past two months?” The difference in the question sentences between 2020 and 2021–2023 occurred because the first emergency declaration in Japan was made in April 2020. Respondents who answered “Yes” to this question were treated as “refraining from regular medical visits for hypertension.”

### Covariates

Several covariates were selected based on previous studies and clinical findings [2, 8–11, 18, 20, 22, 24–27]. The covariates included in the analysis were sex (male or female), age, marital status (married or unmarried), the number of household members, i.e., household size (1 or 2, ≥3), body mass index (BMI) (<18.5, 18.5–24.9, 25.0–29.9, ≥30.0), current alcohol intake (yes or no), current smoking (yes or no), history of diabetes mellitus (yes or no), and history of cardiovascular disease (yes or no). The SES factors other than income, including educational attainment (college or higher, other) and employment status (employer or self-employed or regular employee, non-regular employee, or unemployed), and fear of COVID-19 (low or high), which was considered important during the COVID-19 pandemic, were also included as covariates. We used the Fear of COVID-19 scale (FCV-19S) to assess anxiety and fear of COVID-19 [28, 29] and consists of seven questions, with higher total scores indicating greater fear of COVID-19. Total scores of 7–19 were classified as low COVID-19 fear and 20–35 as high COVID-19 fear, based on the median score of the study participants [20]. We used the covariates measured in 2020. There were no missing values due to the survey design (if any item was not responded to, the survey could not be completed). Additionally, there were no “unknown” options for variables other than annual household income.

### Statistical analysis

To correct the selectivity of Internet-based samples, we used an inverse probability weighting method throughout the analysis (IPW-weighting) [30]. Weights (the inverse of the propensity score, which represents the estimated probability of participating in the survey) were calculated by fitting a logistic regression model using sociodemographic and health-related characteristics to adjust for differences in respondents between this Internet survey and a widely used nationally representative survey from the 2016 Comprehensive Survey of Living Conditions of People on Health and Welfare [31]. The details of the calculation for IPW are described in previous studies [19, 32].

Sociodemographic and health-related characteristics of study participants were calculated according to equivalized annual household income in 2020. The corresponding values after IPW-weighting were also calculated. Differences between the groups were compared by assessing the standardized mean differences (SMD) which assess the standardized absolute difference by considering standard deviations [33]. An SMD of ≥|0.25| was set as the cut-off point determining the large group difference in this study [33].

An IPW-weighted multivariate robust Poisson regression analysis was performed to estimate the proportion of those refraining from regular medical visits in 2020 for each equivalized annual household income, and their proportion ratios (PRs) with the higher-income group as the reference. Model 1 was adjusted for sex, age, marital status, household size, BMI, current smoking, current alcohol intake, history of diabetes mellitus and cardiovascular disease, and fear of COVID-19. Model 2 was additionally adjusted for educational attainment and employment as other SES factors. In addition, to explore whether the association between equivalized annual household income and refraining from regular medical visits varied by respondent characteristics, subgroup analyses by sex, age (<60 years and ≥60 years), household size (1 or 2 and ≥3), and employment status (employed and unemployed) were conducted as sensitivity analyses. Interactions between equivalized annual household income and these factors were tested, excluding the unknown income group. Furthermore, in the subgroup with a pronounced association between equivalized annual household income and refraining from regular medical visits, we used the quartiles of equivalized annual household income as the exposure variable to examine the detailed association. Then, we calculated the *P* for trend, excluding the unknown income group.

We then assessed the time series change in the proportion of those refraining from regular medical visits using a Poisson generalized linear mixed model for repeated measurements with autoregressive order 1 (AR (1)) correlation structure. AR (1) is a standard method for assessing the covariance matrix in mixed model analyses of longitudinal data [15, 34]. The results were also adjusted for the same covariates used in Model 2, as previously mentioned. The proportion of those refraining from regular medical visits was compared to that of other groups in each year, using the respective higher-income group as a reference.

All data were analyzed using SAS software (version 9.4 M4; SAS Institute, Cary, North Carolina, USA). Statistical significance was set at *p* < 0.05. Bonferroni correction was applied to adjust for multiple comparisons when assessing changes in the proportion of refraining from regular medical visits during 2020–2023, stratified by equivalized annual household income. Continuous variables are expressed as the mean ± standard deviation. Absolute numbers and percentages were used to present the categorical variables.

## Results

### Baseline characteristics

After IPW-weighting, the mean age was 64.8 ± 10.3 years and 63.7% of participants were men, the median annual household income was 4,500,000 yen, and the median equivalized annual household income was 3,182,000 yen. Compared with the higher-income group (*n* = 933), the lower-income group (*n* = 1363) was older and had higher proportions of participants with household size ≤ 2, lower BMI, lower education, unemployed, and higher fear of COVID-19 (Table 1).

**Table 1:** Sociodemographic and health-related characteristic of study participants by equivalized annual household income in 2020

	Income category by equivalized annual household income						Standardized mean differences in IPW-weighted values		
	Lower (*n* = 1363)		Higher (*n* = 933)		Unknown (*n* = 536)				
Variables	Crude	IPW-weighted	Crude	IPW-weighted	Crude	IPW-weighted	Lower vs higher	Lower vs unknown	Higher vs unknown
Men, %	61.6	66.4	74.0	62.9	54.1	58.6	0.15	0.20	0.06
Age, years	67.2 ± 9.7	66.8 ± 10.0	61.5 ± 10.8	62.5 ± 10.5	65.0 ± 9.5	64.3 ± 9.5	0.39	0.27	−0.14
Married, %	75.1	81.9	78.4	82.0	72.6	69.9	0.03	0.29	0.26
Household size, %									
1 or 2 persons	74.5	74.5	58.3	60.1	61.2	58.4	0.26	0.36	0.09
≥3 persons	25.5	25.5	41.7	39.9	38.8	41.7	−0.26	−0.36	−0.09
BMI, %									
<18.5 kg/m^2^	2.9	7.0	1.6	0.6	4.1	2.2	0.32	0.20	−0.14
18.5–24.9 kg/m^2^	63.4	56.3	53.9	45.6	58.8	60.0	0.23	−0.08	−0.31
25.0–29.9 kg/m^2^	26.9	27.8	34.6	45.8	30.2	30.8	−0.37	−0.03	0.34
≥30.0 kg/m^2^	6.9	8.9	9.9	8.1	6.9	7.0	0.02	0.05	0.03
Smoking, current, %	14.8	12.8	23.4	19.5	16.8	15.5	−0.19	−0.09	0.11
Alcohol intake, current, %	57.4	53.3	67.4	61.1	53.9	48.3	−0.12	0.08	0.20
Educational attainment, %									
College or higher	40.7	27.3	56.2	44.8	39.2	29.9	−0.45	−0.07	0.38
Others	59.3	72.7	43.8	55.3	60.8	70.1	0.45	0.07	−0.38
Employment, %									
Employer/self-employed/ regular employee	16.1	16.0	55.6	46.2	23.7	25.4	−0.63	−0.21	0.41
Non-regular employee	15.1	19.4	13.1	18.1	15.7	15.1	−0.01	0.08	0.09
Unemployed	68.8	64.7	31.3	35.7	60.6	59.6	0.58	0.11	−0.46
Fear of COVID-19, %									
Low	45.1	42.1	55.2	60.8	43.7	42.0	−0.37	0.04	0.41
High	54.9	57.9	44.8	39.2	56.3	58.0	0.37	−0.04	−0.41

IPW-weighting was used to correct the selectivity of Internet-based samples

Age was presented as mean ± SD

*IPW* inverse probability weighting, BMI body mass index, SD standard deviation

### Association between equivalized annual household income and refraining from regular medical visits for hypertensive patients in 2020

The IPW-weighted proportion of hypertensive patients refraining from regular medical visits in 2020 was 19.6% in the lower-income group, 8.8% in the higher-income group, and 8.7% in the unknown-income group. The proportion of those refraining from regular medical visits in 2020 was significantly higher in the lower-income group compared with the higher-income group even after further adjustments for SES factors including educational attainment and employment status (Table 2). There was no significant difference in the proportion of those refraining from regular medical visits between the high-income group and the unknown-income group (Table 2).

**Table 2:** Association between equivalized annual household income and refraining from regular medical visits for hypertensive patients in 2020

	Weighted proportion of hypertensive patients refraining from regular medical visits^a^ (%)	Adjusted PR (95% CI) of	
		hypertensive patients refraining from regular medical visits	
Strata		Model 1	Model 2
Income category by			
Equivalized annual household income			
Lower	19.6	1.86 (1.13–3.06)	2.03 (1.25–3.30)
Higher	8.8	1.00 (Reference)	1.00 (Reference)
Unknown	8.7	0.89 (0.52–1.52)	1.00 (0.55–1.82)

We weighted the Poisson regression models using IPW to correct the selectivity of Internet-based samples

Model 1 was adjusted for sex, age, marital status, household size, BMI, current smoking, current alcohol intake, history of diabetes mellitus and cardiovascular disease, and fear of COVID-19. Model 2 was additionally adjusted educational attainment and employment

PR proportion ratio, CI confidence interval, IPW inverse probability weighting, BMI body mass index

^a^Weighting was performed using IPW, but covariates were not adjusted for as they were in Model 1 or Model 2

### Change in proportion of refraining from regular medical visits by equivalized annual household income for hypertensive patients

**Fig. 1:**
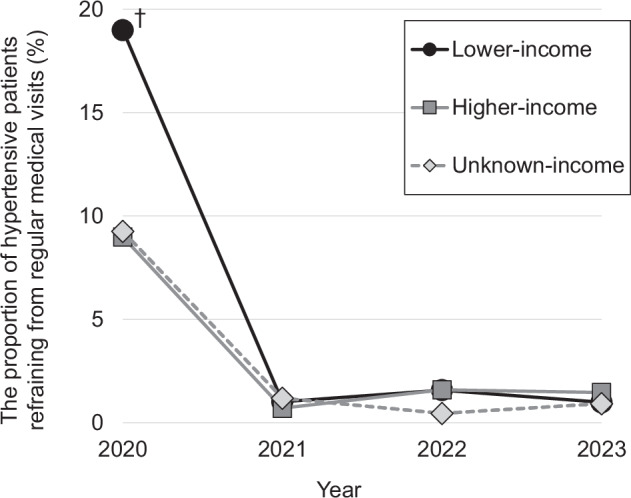
Change in proportion of refraining from regular medical visits by equivalized annual household income for hypertensive patients. Analyses were weighted using IPW to correct the selectivity of Internet-based samples. They were adjusted for sex, age, marital status, household size, BMI, current smoking, current alcohol intake, history of diabetes mellitus and cardiovascular disease, fear of COVID-19, educational attainment and employment. The proportion of those refraining from regular medical visits was compared to that of other groups, using the respective higher-income group for each year as a reference. Bonferroni-corrected *p* < 0.00625 was considered statistically significant. IPW inverse probability weighting, BMI body mass index. ^†^Bonferroni-corrected *p* < 0.00625

The proportion of hypertensive patients refraining from regular medical visits in 2020 was significantly higher in the lower-income group compared with the higher-income group after IPW-weighting and adjusting for all covariates (Fig. 1). However, from 2021 onwards, the proportion of those refraining from regular medical visits has declined sharply in all income groups. There were no significant differences in the proportion by income group in any subsequent year (Fig. 1).

### Sensitivity analyses

When the participants were stratified by sex, adjusted PR in the lower-income group was higher for women (adjusted PR for women and men: 3.14 and 1.21, respectively) with a significant interaction between sex and equivalized annual household income (*P* for interaction = 0.016) (Table 3). After categorizing women into the quartiles of equivalized annual household income, the second quartile income group had a significantly higher adjusted PR compared with the fourth quartile, and a significant *P* for trend was observed (*P* for trend = 0.0095) (Table 4). No significant interactions were observed between the equivalized annual household income and the groups by age (<60/≥60 years), household size (1–2/≥3), and employment status (employed/ unemployed) on the proportion of hypertensive patients refraining from regular medical visits (Table 3).

**Table 3:** Subgroup analyses by sex, age, household size, and employment status in 2020

	Adjusted PR (95% CI) of			
	Hypertensive patients refraining from regular medical visits			*P* for interaction^a^
	Income category by equivalized annual household income			
Strata	Lower	Higher	Unknown	
Sex				
Men	1.21 (0.75–1.93)	1.00 (Reference)	0.84 (0.48–1.47)	0.016
Women	3.14 (1.44–6.82)	1.00 (Reference)	1.26 (0.46–3.45)	
Age				
<60	1.39 (0.87–2.20)	1.00 (Reference)	0.76 (0.39–1.47)	0.28
≥60	2.38 (1.24–4.58)	1.00 (Reference)	1.22 (0.53–2.79)	
Household size				
1 or 2	1.92 (1.02–3.60)	1.00 (Reference)	1.33 (0.62–2.84)	0.76
≥3	2.12 (1.23–3.65)	1.00 (Reference)	0.57 (0.28–1.15)	
Employment				
Employed	2.14 (1.26–3.65)	1.00 (Reference)	0.92 (0.46–1.82)	0.88
Unemployed	1.75 (0.92–3.31)	1.00 (Reference)	1.11 (0.47–2.65)	

We weighted the Poisson regression models using IPW to correct the selectivity of Internet-based samples. The results were adjusted for the same covariates as Model 2 in Table 2

*PR* proportion ratio, *CI* confidence interval, *IPW* inverse probability weighting, *BMI* body mass index

^a^The P for interaction was calculated after excluding the unknown-income group

**Table 4:** Association between women’s equivalized annual household income quartiles and refraining from regular medical visits among hypertensive women in 2020

	Adjusted PR (95% CI) of hypertensive women refraining from regular medical visits	*P* for trend^a^
Strata		
Income category by women’s equivalized annual household income		
First quartile (Lowest)	1.92 (0.79–4.66)	
Second quartile	6.07 (2.52–14.63)	0.0095
Third quartile	1.42 (0.44–4.62)	
Fourth quartile (Highest)	1.00 (Reference)	
Unknown	1.41 (0.52–3.88)	

We weighted the Poisson regression models using IPW to correct the selectivity of Internet-based samples. The results were adjusted for the same covariates as Model 2 in Table 2. The first quartile income group, below 2,475,000 yen; the second quartile income group, 2,475,000–3,182,000 yen; the third quartile income group, 3,183,000–4,900,000 yen; the fourth quartile income group, at or above 4,901,000 yen; and an unknown-income group

*PR* proportion ratio, *CI* confidence interval, *IPW* inverse probability weighting, *BMI* body mass index

^a^The *P* for trend was calculated after excluding the unknown-income group

## Discussion

This study revealed that lower equivalized annual household income was associated with refraining from regular medical visits for hypertensive patients in 2020 during the COVID-19 pandemic. This association remained unchanged after adjustment for various covariates, including COVID-19 fear, suggesting that economic status may have led to refraining from regular medical visits during the COVID-19 pandemic. Meanwhile, from 2021 onwards, the proportion of hypertensive patients refraining from regular medical visits was considered limited, with no observed differences between the groups based on equivalized annual household income.

Our study found that income-related inequities in medical care utilization for hypertensive patients could have existed in Japan under social restrictions due to the COVID-19 pandemic. Previous studies have reported that lower household incomes were associated with an increased risk of delaying or refraining from medical care during the COVID-19 pandemic [10, 11]. For instance, a US online survey found that during the pandemic, individuals with the lowest household incomes had a 1.5 times higher risk of delaying medical care than those with the highest household incomes [11]. A study using data from a large group of Japanese hospitals showed that in 2020, during the COVID-19 pandemic, there was a 23% year-on-year decrease in first outpatient visits and a 9% decrease in outpatient revisits [4]. The previous reports from the JACSIS suggested the involvement of lower household income in refraining from regular medical visits during the pandemic; this previous study included patients with various conditions including diabetes or respiratory diseases and has not examined the long-term change in the medical visit status after 2021 [10]. Meanwhile, the present study, which focused on hypertensive patients, suggests that lower equivalized annual household income linked to higher risk of refraining from regular medical visits, potentially leading to poorer blood pressure control during the COVID-19 pandemic. Social restrictions due to infectious diseases such as COVID-19 or major disasters may occur again in the future. Appropriate public measures such as providing effective financial support and promoting telemedicine using digital devices, which can improve access to medical care, may help reduce income-related health inequities in these crises [17].

Previous studies have reported several constraints that contribute to refraining from medical visits. These include financial constraints, such as “cost,” time-related constraints, such as “long waiting times” or “being too busy,” geographical constraints, such as “no nearby hospitals” or “lack of transportation,” and psychological constraints, such as “dislike of hospitals or doctors,” often in combination [9, 35]. Among these constraints, “financial constraints” and “time-related constraints” have been reported to underlie the association between lower household income and refraining from medical visits [35]. Social restrictions due to the COVID-19 pandemic may have led to unemployment, reduced income, and worsening employment conditions, resulting in refraining from regular medical visits due to these factors among hypertensive patients with lower household incomes.

The study also showed that the association between household income and refraining from regular medical visits disappeared from the 2021 survey onwards, when social restrictions in Japan began to ease, including the lifting of the state of emergency declaration. This finding is consistent with a UK study using COVID-19 survey data, which found that initial income-related inequity in general practitioner visits in April 2020 lessened over time as pandemic measures advanced [36]. Although there are differences in the severity of social restrictions and healthcare systems between Japan and the UK, our results suggest that government-imposed social restrictions have a considerable impact on medical care utilization, especially for patients with lower household incomes.

The risk of refraining from regular medical visits in the lower-income group was higher for women, which is consistent with previous studies [10, 22]. Moreover, a significant interaction between sex and equivalized annual household income suggests that women may be at greater risk of income-related inequities in medical care utilization. Financial and time-related constraints have been reported as primary reasons for refraining from medical visits [35]. Several reports have also shown that women spent significantly more time on housework and childcare than men during the COVID-19 pandemic [37, 38]. Consequently, women with lower household incomes may have been more likely to refrain from regular medical visits due to the combined pressures of financial constraints and greater time-related constraints from spending more time on housework and childcare. Furthermore, the significant *P* for trend across the quartiles of equivalized annual household income implies that women tend to refrain from regular medical visits as their income levels decrease. Meanwhile, the highest proportion of women refraining from regular medical visits was observed in the second quartile income group, not in the lowest. One possible explanation is that some participants in part of the lowest income quartile group may have received public assistance, such as medical subsidies, or may have been financially stable due to their assets or savings.

This study has several limitations. First, all the information used in this study, such as the diagnosis of hypertension, annual household income and refraining from regular medical visits, was self-reported, so the possibility of misclassification was inevitable. It is unclear whether the outcome of refraining from regular medical visits was due to patients’ own decisions or to restrictions on medical visits imposed by medical providers. Including the latter in the outcome may weaken the association between equivalized annual household income and refraining from regular medical visits. The study also did not include specific blood pressure values, making it impossible to assess whether hypertension had worsened based on objective indicators. Additionally, the information on public assistance or financial assets was not collected. Second, this study does not have data before 2020, making it impossible to assess the association between equivalized annual household income and refraining from medical visits for hypertensive patients prior to the COVID-19 epidemic. Third, ~20% of the participants did not report their income and were categorized as the unknown-income group. It is generally observed that up to one-third of respondents in large population-based surveys have missing income information [39]. Previous research suggested that participants who did not report their income were often similar to individuals with lower household incomes [40, 41]. However, in our study, the proportion of participants refraining from regular medical visits in the unknown-income group was similar to that in the higher-income group. This may be because our study collected by the anonymized Internet survey in Japan [40]. Finally, because we used data from an Internet survey, the sample may not fully reflect the demographic distribution of the general population. It has been reported that individuals with lower household incomes have limited Internet access [42]. However, while specific data on the income of hypertensive patients is unavailable, the median annual household income of participants in this Internet survey did not show a marked difference from that of the general Japanese population in 2020 [43]. To address these limitations, future studies should include objective data on income, medical care utilization, and blood pressure levels, and evaluate the impact of refraining from regular medical visits on blood pressure.

## Conclusion

Using a large-scale, nationwide Internet survey in Japan, we found that hypertensive patients with lower equivalized annual household incomes were significantly more likely to refrain from regular medical visits in 2020 during the COVID-19 pandemic. The exacerbation of hypertension in such vulnerable patients by refraining from regular medical visits should be noted, especially when social restrictions are imposed. Further strategies to reduce income-related inequities in medical care utilization, such as providing effective financial support and promoting telemedicine, may be necessary to better prepare for future public health crises.
